# Cochlear anatomy study used to design surgical instruments for cochlear implants with two bundles of electrodes in ossified cochleas

**DOI:** 10.1016/S1808-8694(15)31088-0

**Published:** 2015-10-19

**Authors:** Mariana Bogar, Ricardo Ferreira Bento, Robinson Koji Tsuji

**Affiliations:** 1 Medical Student; 2 Full Professor of Otorhinolaryngology - Medical School of the University of São Paulo, Head of the Department of Ophthalmology and Otorhinolaryngology of the FMUSP; 3 Graduate student in Otorhinolaryngology - PHD - Medical School - University of São Paulo, Assistant Physician - USP University Hospital. Faculdade de Medicina da Universidade de São Paulo

**Keywords:** internal carotid artery, ossified cochlea, double array, cochlear implant

## Abstract

Cochlear ossification, mainly secondary to meningitis, prevents the complete conventional cochlear implant insertion. Implants with two electrode bundles shorter than the conventional ones were specifically developed for ossified cochleas. However, during surgery there is a high risk of damaging the internal carotid artery (ICA). Therefore, measuring cochleostomy depth in order to insert the two electrode bundles would greatly increase the procedure's safety.

**Aims:**

1) Find the distances between cochleostomies and ICA in cadaver temporal bones. 2) Design an instrument that can be used in cochlear implant surgery to introduce an implant with two bundles of electrodes.

**Study Design:**

Experimental prospective.

**Materials and Methods:**

In 21 temporal bones from cadavers we performed: 1) canal wall down mastoidectomy; 2) cochleostomy in the cochlear basal and middle turns; 3) ICA identification; 4) Length determination between the cochleostomies and the artery.

**Results:**

the average distance ± standard deviation obtained for the upper tunnel was of 8.2 ± 1.1 mm and for the lower tunnel it was of 8.1± 1.3 mm. The shortest distance found was of 6.5 mm for the upper tunnel and 6.0 mm for the lower tunnel.

**Conclusion:**

Despite the values calculated, we concluded that the best value to be considered in creating a surgical instrument are the minimum lengths obtained for each one of the cochlear turns, because this is the safest way to avoid damaging the ICA, that can be fatal.

## INTRODUCTION

Profound hearing loss is an impairment that prevents the individual from properly communicating and from intellectual development. Thus, hearing loss has a marked impact on the life style and personality development of the individual with hearing impairment^1^; it bears congenital and acquired etiologies and, according to the World Health Assembly (WHA), its world prevalence in 1995 was of 2.2%, affecting 120 million people. In Brazil, it is very difficult to estimate the incidence of hearing impairment, because it is not a disease one is obligated to report to public health agencies.^2^

Conventional hearing aids amplify the sounds present in the environment. Such device is used to treat numerous types of hearing loss; however, depending on the degree of hearing dysfunction, it has limited results. When a patient does not reach a sound discrimination level above 40% in phrases recognition tests in an open field with the best possible hearing aid, the cochlear implant becomes an alternative.^3^

The cochlear implant is an electrical stimulator made up of an external speech processing unit and one internal support and programming unit, made up of a reception antenna and stimulation electrodes.^4^ The implant allows not only for hearing, but also de recognition of speech sounds. It works as the entire ear, it captures the sound, decodes the messages and sends them to the brain through electrodes, replacing the organ of Corti and directly stimulating the nerve fibers and the ganglionary cells of the auditory nerve.^5^

The conventional procedure to place the cochlear implant is carried out through the transmastoid approach, with posterior tympanoplasty, followed by cochleostomy (opening the labyrinth block with a perforating burr).^6^

After the 90's, multichannel cochlear implants have been established as surgical treatment for sensorineural hearing impairment.^5^ Among the causes for sensorineural hearing loss, meningitis stands out as the major source for the acquired impairment^7^. Since it is endemic in Brazil, its prevalence is higher when compared to North America and Europe, reaching values of 8% among all the causes for profound hearing loss.^8^ The post-meningitis hearing loss is characterized for being severe or profound, bilateral and sensorineural.^9^ Pneumococci meningitis has the worst auditory prognosis and can cause up to 30% of permanent hearing loss in survivors.^10^ In 80% of these patients, the hearing loss is associated with cochlear ossification.^11^ Besides cochlear ossification, cochleovestibular nerve degeneration has also been described in some cases.

Cochlear ossification is a sequela that can be caused by trauma to the temporal bone, otosclerosis and chronic otitis media; however, the main cause is meningitis.^12^ The ossification process that stems from these disorders usually starts near the round window and moves up to the apex. Therefore, the basal turn tympanic ramp usually is the most involved portion.^6^

It is known that total or partial obliteration of the initial portion of the cochlear basal turn prevents the complete insertion of the electrodes used in conventional cochlear implants.^13^ This represents a major problem, because studies based on data from 327 patients have proven that the greater the insertion, and consequently the higher the number of electrodes connected, better is the speech recognition index.^14^ Thus, many techniques have been used and tested in order to deeply implant increasingly more electrodes in ossified cochleas. However, none of these techniques have allowed full electrode insertion preserving the cochlear anatomy.^15^, ^16^, ^17^

In 1993, Cohen and Waltzman proposed the removal of the newly formed bone from the beginning of the cochlear basal turn.^15^ Gantz and et al. suggested the complete removal of the basal turn.^16^ In 1997, Balkany modified the technique described by Gantz and preserved the initial portion of the cochlear basal turn.^17^ Nonetheless, in these cases there were post-operative problems with the stimulation of the auditory nerve, such as pain and discomfort, and the incomplete insertion of intracochlear electrodes.

In 1997, Lenarz et al., proposed the insertion of two parallel lines of electrodes in separate canals created in the basal and medium cochlear turn, thus developing the cochlear implant with two bundles of electrodes.^18^ One major advantage of this implant is that the medium turn is clear in approximately 50% of the cases described as total cochlear obliteration. Moreover, this technique reduces the risk of damaging the facial nerve. The cochlear implant with 2 bundles of electrodes was especially created for totally obliterated cochleas or for those surgically inaccessible.

In cochlear implants with two bundles of electrodes, the surgeon must also perform a mastoidectomy and a posterior tympanotomy. Following that, a cochleostomy is made antero-superiorly to the cochlear basal turn, opening access to the scala tympani. Another cochleostomy is carried out in the second cochlear basal turn. This second cochleostomy is carried out caudally to the cochleariform process, at 2mm anterior to the oval window.

In patients with total obliteration of the scala tympani, after having performed the first cochleostomy, the scala vestibularis must be exposed. Should it also be ossified, the basal turn is drilled from the cochleostomy all the way to the anterior wall of the cochlea. The newly formed bone is always white, and so it can be distinguished from the cochlear bone, which is yellowish and harder. Stop drilling as soon as you reach the anterior cochlear wall bone in order to avoid damaging the internal carotid artery, which is closely related with the anterior portion of the cochlea. However, for such a risky procedure, this description is very inaccurate. Lenarz et al.^13^ estimated the distance from the first cochleostomy all the way to the anterior cochlear wall in the basal turn to be of 8 to 11mm. Now, the distance from the second cochleostomy, again to the second cochlear turn is of 5 to 6mm. However, in 2002, the same author reports that in a second study, with implanted patients with two bundles of electrodes, the more profound insertion of electrodes in the basal turn was of 10.3mm and in the second cochlear turn, this distance was of 8.2mm.^19^

In 10 procedures carried out in 2005, the surgeon had difficulties in performing the cochleostomy with conventional drills in these ossified cochleas. In 3 cases the drill broke upon partial penetration.

Because of the important relation between the carotid artery and the cochlea, and the associated anatomical variations, as well as the severity of an eventual injury to this vessel, there came the idea of measuring the distance from the cochleostomy to this artery in cadaver bones in order to determine the maximum depth that the surgeon can reach.

The goals of the present investigation are:

1. To measure the distance from the cochleostomy to the internal carotid artery in the basal and middle turns of the cochleas from cadavers' temporal bones.
2. Based on these measures, to build metered instruments that may be used not only to guide the surgeon as to the depth reached, but also to be used as a manual drill.

## MATERIALS AND METHODS

We legally obtained 21 temporal bones from adult cadavers. All the bones were formalized (70% Formaldehyde) and dissected using the high speed motor (40.000 rpm) from Volvere (Japan). We performed mastoidectomy in these bones, removing all the cortical bone from the mastoid and that from the posterior wall of the external acoustic meatus, in such a way as to expose the cochlear promontory in the middle ear and the two windows (oval and round), and the cochleariform process. The malleous and incus were removed, and only the stapes remained in position.

Before performing the cochleostomy, we continued to drill in order to dissect and identify the portion of the carotid artery closest related with the cochlea.

**Figure 1:**
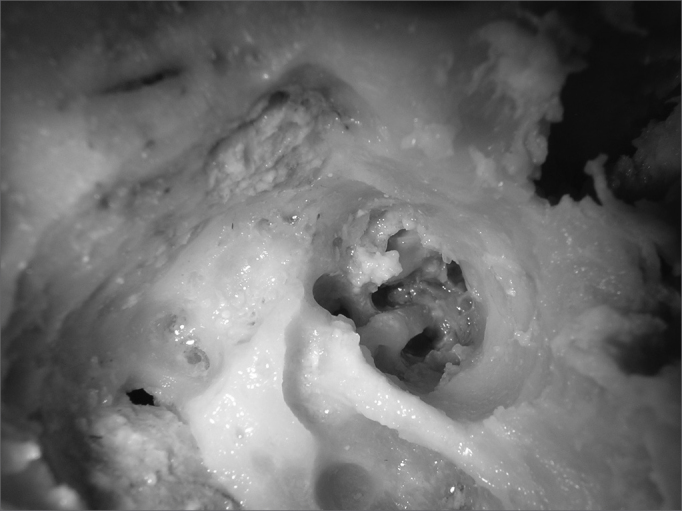
Superior and inferior tunnels, joining the cochleostomy to the internal carotid artery.

**Figure 2:**
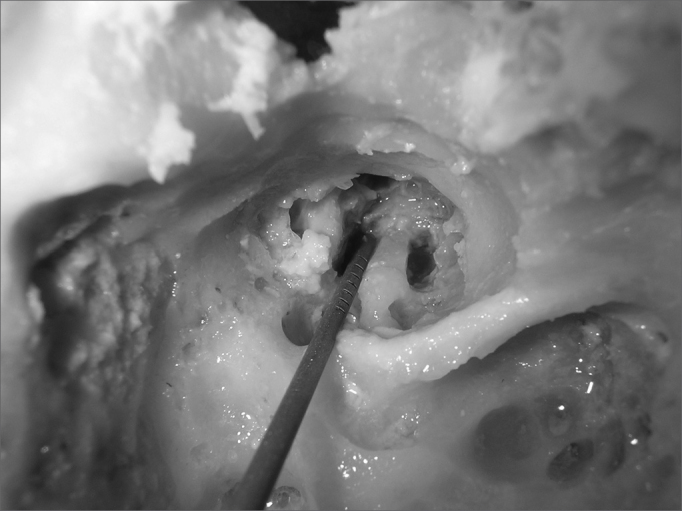
Doing the measurements using the instrument graded in millimeters.

After identifying all the structures mentioned, we carried out the first cochleostomy antero-superiorly to the round window, in the cochlea basal turn. The second cochleostomy was carried out caudally to the cochleariform process, and at 2mm anteriorly to the cochlea's middle turn. After the cochleostomy, we drilled a communication (Tunnel) between them and the internal carotid artery. The burr used had the same thickness of the graded measuring device, so that it could fit precisely in order to carry out the measurings. The upper portion of the cochlea was removed, so that we could see the measuring device going through the cochlear turns from the cochleostomy and reaching the internal carotid artery (Figure 1). This procedure does not alter the values of the measures. The measuring was carried out from the inferior border of the cochleostomy entrance, all the way to the greater posterior projection of the internal carotid artery (Figure 2).

The numeric variables were discussed by means of their averages ± standard deviation, confidence interval of 95% (CI 95%) and minimum and maximum values. Since this is a descriptive study of initial exploration, the sample was not calculated, and we used a convenience sample, according to the availability of specimens to dissect during the study.

We made a straight punctioning device, the shape of a six-angled tip from dull stainless steel, graded from 1.0 in 1.0mm by order to the Azelindo Mercansoli EPP Company, from Jundiaí- SP. These punctioning instruments measure 16cm in length. The initial diameter at their tips is of 1.0mm, and they thicken gradually until they reach 1.3 mm next to the handle.

The Ethics Committee protocol No. is 371/2005.

## RESULTS

The average measure ± standard deviation for the superior tunnel was 8.2 ± 1.1 mm

The average measure ± standard deviation for the inferior tunnel was 8.1± 1.3 mm.

The shortest distance found was 6.5 mm for the superior and 6.0 mm for the inferior.

**Table 1:** Measures of the distances between the internal carotid artery and the cochleostomy performed in the basal turn (inferior tunnel) and the middle turn (upper tunnel) of the cochlea.

Specimen	Length of the inferior tunnel (mm)	Length of the superior tunnel (mm)
1	10	9
2	9	9
3	10	9
4	8	9
5	6,5	8
6	8	8
7	6,5	6,5
8	7	7
9	7	8
10	8	10
11	10,5	10
12	8	8
13	9	8
14	7	8
15	9	10
16	9	8
17	6	7
18	9	8
19	7	6,5
20	7	7
21	8	9

**Table 2:** Mean values of the distances ± standard deviation, 95% confidence interval (CI 95%) of the average, minimum and maximum values of the inferior and superior tunnels.

	Mean ± standard deviation	95% CI from the average	Minimum value	Maximum value
INFERIOR TÚNNEL	8,1 ± 1,3	7,4-8,6	6,0	10,5
SUPERIOR TÚNNEL	8,2 ± 1,1	7,7-8,7	6,5	10

The average measure ± standard deviation for the superior tunnel was 8.2 ± 1.1 mm

The average measure ± standard deviation for the inferior tunnel was 8.1± 1.3 mm.

The shortest distance found was 6.5 mm for the superior and 6.0 mm for the inferior.

## DISCUSSION

The cochlear implant (CI) is known as an effective method to treat bilateral profound sensorineural hearing loss^4^^,^^17^^,^^20^. The cases of ossified cochleas continue to be a major challenge for surgeons, because it is not possible to use all the electrodes from the modern cochlear implant devices, thus making results much worse than those obtained from cochleas without ossification. Other factors may also be responsible for these bad results, such as alterations in the synapses of the cochlear branch of the cochleovestibular nerve, which reduce the conduction of nerve impulses generated by the CI. The surgical techniques for CI were developed after an intense anatomical study of the temporal bone. The surgeon must have a profound knowledge about inner ear anatomy, especially concerning the cochlea in order to be successful^6^^,^^21^. It is known that the carotid artery is very near the cochlea at the junction where its vertical and horizontal segments merge.^22^ However, anatomy books and atlases on this region do not bear complete and detailed information on the relation of the small structures that make up the inner ear, such as the cochlea and the internal carotid artery, which are extremely important for the CI surgery to implant two-bundle electrodes.

Although there are some studies^22^^,^^23^ which aim at studying the distances and relations between these two structures, the measures obtained are from the external wall of the cochlear turns to the artery, and such measures by themselves do not add relevant information to be considered during a two-bundle electrodes CI surgery. In such procedure it is important to know the precise safe distance than one can drill from the cochleostomy towards the anterior cochlear wall, so as to avoid damaging the ICA. This drilling is made so that a larger number of electrodes can be implanted, because in such cases the cochleas are partially or totally ossified.^21^

In our study, we decided to simulate part of what is done during the two-bundle electrodes CI surgery, making the cochleostomy in the cochlear turns of the temporal bones. From these cochleostomy we continued drilling through the cochlea and reached the ICA. Thus, we were able to measure the maximum distance that one can drill without injuring the artery.

Therefore, the measures found in our study can not compare to the ones from previous studies^20^^,^^22^^,^^23^; because, besides being different measures, the angle at which these measures were taken is also different. We based our study on the technique used during the surgical procedure, maintaining such angle, in the other studies these measures were obtained by using an angle that will produce the minimum distances between these structures.

We must stress that these specimens were obtained from the city morgue, without information about gender and race. The exact age at the time of death was also unknown; however we knew that all these temporal bones came from adult cadavers. Nonetheless, the importance of these data is debatable, because the key structures such as the cochlea, the middle ear, ossicles and tympanic membrane are already established at birth^6^. Thus, the surgical technique used for CI in children is very similar to the one used in adults^5^. However, despite the literature stating that the inner ear structures are fully developed at birth, keeping the adult sizes^24^^,^^25^^,^^26^ in a study that analyzes the differences of cochlear turns and carotid canal in two groups of individuals broken down by age (Group I up to 4 years of age and Group II made up of those individuals above 4 years of age), the author states that in Group I these distances were significantly shorter.^22^ Nonetheless, the Group I sample had only 12 temporal bones, and this could statistically jeopardize their results. Therefore, other studies should be undertaken in order to guarantee if the distances attained in our study could also be applied for the two-bundle electrodes CI surgery in children.

In regards of gender and side, Penido^23^ states he did not find any significant association between these variables and inner ear anatomy. In the literature we did not find references about the influence of race on these structures.

## CONCLUSIONS

Despite the parameters calculated, we concluded that the best measure to be considered in making the surgical instrument are the minimal distances obtained in each one of the cochlear turns. The ICA injury during surgery is considered almost fatal, because it can cause a difficult-to-control bleeding and bring about severe consequences to the Central Nervous System. Therefore, to work with the minimal measures is the safest way to avoid such problems.

Besides guiding the surgeon as to which depth he/she can drill during cochleostomy without damaging the ICA, we intend to use it as a manual drill, which will work without power, thus not heating up, bearing lower risk of breakage during surgery, as it has happened with some conventional burrs.
